# Spermatozoal cell death-inducing DNA fragmentation factor-α-like effector A (CIDEA) gene expression and DNA fragmentation in infertile men with metabolic syndrome and normal seminogram

**DOI:** 10.1186/s13098-016-0192-y

**Published:** 2016-11-16

**Authors:** Ayman Z. Elsamanoudy, Hussein Abdelaziz Abdalla, Mohammed Hassanien, Mohammad A. Gaballah

**Affiliations:** 1 Department of Clinical Biochemistry, Faculty of Medicine, King Abdulaziz University, Jeddah, Saudi Arabia; 2 Department of Medical Biochemistry, Faculty of Medicine, Mansoura University, Mansoura, Egypt; 3 Department of Dermatology, Andrology and STDs, Faculty of Medicine, Mansoura University, Mansoura, Egypt; 4 Department of Medical Biochemistry, Faculty of Medicine, Tanta University, Tanta, Egypt

**Keywords:** CIDEA, Insulin genes expression, DNA fragmentation, Seminal glucose, Male infertility, Metabolic syndrome

## Abstract

**Background:**

This is the first study to investigate spermatozoal cell death-inducing DNA fragmentation factor-α-like effector A (CIDEA) gene expression and DNA fragmentations in the spermatozoa of men diagnosed with metabolic syndrome (MS) who have normal seminograms with unexplained infertility, and to correlate these parameters with seminal glucose concentration.

**Methods:**

This study included 120 participants: 75 male subjects with MS (38 fertile and 37 infertile), and a control group of 45 fertile males without MS. HOMA-IR, semen analysis, and biochemical measurement of seminal plasma insulin and glucose levels were carried out. Spermatozoal insulin gene and CIDEA gene expressions were performed by the RT-PCR method. The percentage of spermatozoal DNA fragmentation was also estimated.

**Results:**

The spermatozoal insulin and CIDEA gene expression, as well as the DNA fragmentation, were significantly higher in the infertile MS group than in the fertile MS group, and significantly higher in both the MS groups than in the control group. Seminal glucose concentration showed significant positive correlations with seminal insulin level, spermatozoa insulin, CIDEA gene expression, and DNA fragmentation. Moreover, there was a positive correlation between spermatozoa CIDEA gene expression and DNA fragmentation.

**Conclusions:**

It can be concluded that MS may affect male fertility at the molecular level, through its possible inducing effect of spermatozoa CIDEA and insulin gene expression, DNA fragmentation, and increased seminal glucose.

## Background

Infertility is defined by the World Health Organization (WHO) as “the inability of a couple to achieve conception or bring a pregnancy to term after 12 months or more of regular, unprotected sexual intercourse” [1]. Infertility affects up to 15% of couples. A male factor is solely responsible in about 20% of infertile couples and contributory in 30–40% of cases [2]. Metabolic syndrome (MS) or syndrome of insulin resistance is a multi-factorial endocrinopathy that is framed by classical cardiovascular risk factors, including insulin resistance, hyperglycemia, abdominal obesity, a pro-inflammatory state, essential hypertension, and dyslipidemia. The frequency of MS is increasing worldwide, and it is suggested to be an important reason for the mortality and morbidity caused by cardiovascular diseases [3].

A possible association between MS and male infertility has been hypothesized [4]. Du Plessis et al. [5] postulated that male obesity and MS are associated with hypogonadism. Leisegang et al. [6] reported that insulin and leptin, which are important regulators of male reproduction via modulation of the hypothalamus-pituitary-testes (HPT) axis, were detected in the seminal fluid of obese infertile men. They suggested that both hormones could be synthesized and secreted by ejaculated spermatozoa through apparent autocrine regulatory functions.

Glucose was also detected in human semen, and it represented about half of the sugar consumed by the ejaculated spermatozoa [7]. Oxidative stress is known to play an important role in various manifestations of MS. Hyperglycemia causes oxidative stress and accelerates the accumulation of advanced glycation end products (AGEs) in the male genital tract, products which are capable of producing, endorsing, and/or increasing oxidative stress and of inducing sperm nuclear DNA (nDNA) damage [8]. Sperm nDNA damage may be present in men with normal or abnormal semen parameters [9]. As significant DNA fragmentation can be present in normozoospermic subfertile men, it may be an important factor in unexplained infertility that appears in approximately 15–30% of infertile couples [10]. The need to introduce the evaluation of sperm DNA quality into the clinical setting has been acknowledged [11]; however, it is not routinely assessed in semen analysis according to WHO guidelines [1].

Cell death-inducing DNA fragmentation factor-α-like effector A (CIDEA) belongs to the CIDE family of proapoptotic proteins [12]. It has a role in lipid metabolism, body weight regulation, and the development of metabolic disorders [13]. In humans, the CIDEA gene is predominantly expressed in white fat cells and inhibits adipocyte lipolysis [14]. It is the most up-regulated gene in human white adipose tissue, following dietary weight reduction [15]. CIDEA transcripts of various lengths are present in different human tissues (e.g., heart, skeletal muscle, brain, placenta, white adipose tissue, and kidney) [12], but there are no reports about CIDEA expression by human spermatozoa.

Based on a search of the scientific literature, it is apparent that the effect of MS on male (in)fertility has not been sufficiently investigated. Therefore, with reference to the impact of MS on male (in)fertility, this study aims to investigate spermatozoa DNA fragmentation, CIDEA, and insulin gene expressions in men diagnosed with MS and unexplained delayed fertility, in spite of normal seminograms. It also aims to correlate these variables with seminal glucose concentration, in an attempt to find a possible pathophysiologic mechanism in such subjects.

## Methods

### Selection of subjects

The study was carried out on 120 participants: 38 fertile men with already diagnosed MS, 37 infertile men with already diagnosed MS, and 45 age-matched fertile volunteers as a control group. The fertile volunteers had offspring during the first year of their respective marriages, and they did not meet the criteria of MS. They were healthy, fertile persons personally known to us, and we explained the idea of the study to them by personal communication. When they were asked to participate they accepted and signed informed consent forms.

The fertile men with MS were referred to the Andrology Unit from the obesity clinic at Mansoura University Hospital, and they were considered fertile because all of them had offspring during the first year of their respective marriages. They were referred to the Andrology Unit as they were already MS patients, and their doctors needed to check the maintenance of their fertility status given the continuation of MS. The infertile subjects had normal seminograms according to WHO [1]. Patients represented a consecutive series of men attending the Andrology Unit at Mansoura University Hospital, from January 2013 to July 2014. All of them had been married for ≥1 year with failed conception in spite of regular unprotected intercourse. Members of infertile couples whose infertility had female factors were excluded. A complete detailed infertility sheet was reviewed with each participant. Complete general and local genital examinations, including digital rectal examinations, were carried out. The selected men had normal serum levels of FSH, LH, testosterone (total and free), estradiol, prolactin, and thyroid hormones.

This study excluded any patient with abnormal semen parameters, including leukocytospermia (>1 × 10^6^/ml), or with obvious infertility risk factors detected from the history, e.g. exposure to gonadotoxins, cigarette smoking, recreational drug use, alcohol consumption, surgery for varicocele, undescended testis, urogenital infections. Likewise, infertility risk factors detected during an examination, such as small sized testes, varicocele, and cryptorchidism were exclusion factors. In addition, any patient with any medical problem other than MS such as tuberculosis, heart disease, renal disease, autoimmune disorders, cancer, liver diseases, endocrine disorders e.g. Cushing disease, known or clinically detected acute or chronic inflammatory conditions not related to MS, or long-term medication such as corticosteroids, was also excluded from the study. Color Doppler ultrasounds (CDU) were carried out to detect subclinical varicocele, and affected patients were excluded in addition to those with palpable varicocele.

Based on the clinical and laboratory data, a diagnosis of MS was made according to the diagnostic criteria outlined by Alberti et al. [16]. A minimum of three of the following five criteria need to be fulfilled to obtain a diagnosis of MS:

1. Waist circumference in Middle East and Mediterranean men of ≥94 cm.
2. Blood pressure: systolic ≥130 mmHg and/or diastolic ≥85 mmHg (or antihypertensive medication).
3. Fasting triglycerides ≥1.69 mmol/l (≥150 mg/dl) or relevant medication.
4. Fasting HDL cholesterol <1.00 mmol/l in males (<40 mg/dl) or relevant medication.
5. Fasting glucose >5.5 mmol/l (≥110 mg/dl).

### Samples collection

Five millilitre fasting blood samples were withdrawn on K_2_EDTA and centrifuged at 7000 rpm to prepare the plasma for measurement of fasting plasma glucose and insulin, for calculation using the homeostasis model assessment of insulin resistance (HOMA-IR). One semen sample was collected from each participant in the Infertility Clinic of the Andrology Unit at Mansoura University Hospital, and referred to the laboratory of the Medical Biochemistry Department, Faculty of Medicine, Mansoura University. All semen samples were collected by masturbation after an ejaculatory abstinence period of 3–5 days.

### Standard semen analysis

The sample was collected into a graduated glass measuring cylinder with a wide mouth. The volume was read directly from the graduations. Seminal fluid was left for 60 min at 37 °C to liquefy. After liquefaction, the sperm count and motility (progressive and total) was assessed using the Motility/Concentration module of the computer assisted semen analysis (CASA) system using MiraLab—Egypt (Mira 9000 Sperm Analyzer CASA software). For analysis, a Trinoculer Microscope with a plain objective lens (Olympus), equipped with phase contrast optics and a heated stage (37 °C), was used.

Morphology was assessed by the preparation of a smear and the application of the spermMac stain staining method (Fertipro, Belgium) as outlined by the WHO [1]. Leukocyte concentration was determined using the peroxidase staining technique according to that of Politch and colleagues [17] and first described by Endtz [18]. Viability was assessed using an Eosin Y stain. A blinded observer scored 100 cells for stain uptake (dead cells) or exclusion (live cells) [19, 20]. Seminal plasma was obtained by subjecting the semen samples after liquefaction to centrifugation at 7000 rpm. The supernatant fluid was separated into aliquots and stored at −30 °C until used to estimate the seminal plasma glucose and insulin levels.

### CIDEA and insulin genes expression

#### RNA extraction

After liquefaction, one ml of each collected sample was taken into tubes containing two ml of RNA later solution (Sigma). Spermatozoa were precipitated by centrifugation (7000 rpm, 10 min, 4 °C), and washed three times by phosphate buffered saline (PBS) to obtain a cell free sperm pellet. Total RNA extraction was carried out from the sperm pellet using TriFast TM reagent (PeqLab. Biotechnologie GmbH, Carl-Thiersch St. 2B 91052 Erlongen, Germany, Cat. No. 30-2010) according to the manufacturer’s instructions. The remaining DNA was removed by digestion with DNase I (Sigma). The concentration of isolated RNA was determined spectrophotometrically by measuring the optical density (OD) at 260 nm (Jenway, Genova Model, UK). Ten microlitre of each sample was added to 990 µl of Diethyl pyrcarbonate (DEPC) treated water, and quantified by measuring the absorbance at 260 nm as RNA yield (µg/ml) = A260 × 40 × 100 (dilution factor) [21]. The purity of the RNA was determined by gel electrophoresis using formaldehyde agarose gel electrophoresis and ethidium bromide staining to show two sharp bands, representing 28S and 18S ribosomal RNA.

#### Reverse transcriptase and synthesis of cDNA

First strand cDNA synthesis from the total RNA was performed using QIAGEN LongRange 2 Step RT-PCR Kit (100) (Germany-Cat. No. 205922): 20 μl reaction mix was prepared by addition of 2 μl of template-extracted RNA from the sperm pellet and 9.8 μl DEPC treated water to a reverse-transcription master mix that contained 4 μl LongRange RT Buffer, 5×, 2 μl dNTP mix (10 mM each), 1 μl Oligo-dT (20 μM), 0.2 μl longrange RNase inhibitor (4 U/μl), and 1 μl longrange reverse transcriptase. The protocol for reverse transcriptase and cDNA synthesis was carried out according to the manufacturer’s instructions by mixing the master mix thoroughly and carefully by vortexing for 5 s, incubating for 50 min at 42 °C, then inactivating the enzyme by heating at 85 °C for 15 min. The synthesized cDNA was stored at −80 °C until utilized for semi-quantitative insulin gene and quantitative CIDEA gene expression, respectively.

#### Semiquantitative RT-PCR for insulin gene expression

##### Amplification and detection

Gene-specific primers were purchased from biosearch technologies (CA 94954-6904 USA); insulin: forward, 5′-GCC TTT GTG AAC CAA CAC CTG-3′; and reverse, 5′-GTT GCA GTA GTT CTC CAG CTG-3′ (261 bp fragment) [20]. The mRNA of β-actin was used as an endogenous external control for RT-PCR: Primers for β-actin to generate a (218 bp) fragment were designed as sense primers, 5′-AAGAGAGGCATCCTCACCCT-3′ and antisense primers, 5′-TACATGGCTGGGGTGTTGAA-3′ [22].

For insulin, the conditions for PCR described by Aquila et al. [20] were followed: denaturation at 95 °C for 1 min, annealing at 62 °C for 1 min, and extension at 72 °C for 2 min (40 cycles) and at 72 °C for 7 min for the final extension. For β actin the conditions were 32 cycles of denaturation at 95 °C for 1 min, annealing at 60 °C, 58 °C for 1 min, and an extension at 72 °C for 1 min, then a final extension at 72 °C for 10 min. Amplification was performed in a thermal cycler (TECHEN TC-312, Model FTC3102D, Barloworld Scientific Ltd. Stone, Stafford Shire St 150 SA, UK).

The PCR products were analyzed by electrophoresis and ethidium bromide staining at 1% for insulin, visualized via a light UV Transilluminator (Model TUV-20, OWI Scientific, Inc. 800 242-5560, France), and photographed under fixed conditions (the distance, the light, and the zoom). The resulting photos were analyzed with Scion Image^®^ release Alpha 4.0.3.2. Software for Windows^®^, which performs band detection and conversion to peaks. The areas under each peak were calculated in square pixels and used for quantification. Gene expression levels were determined by calculating the ratio between the square pixel values of the target gene in relation to the internal housekeeping control gene (β actin), minus RT the controls permitted to rule out genomic contamination. Similarly, no products were detected when the RT-PCR step was carried out with no added RNA, indicating that all reagents were free of target sequence contamination.

#### Real time RT-PCR for CIDEA gene expression

##### Gene-specific PCR primers

Primers of CIDEA for real time RT-PCR according to Yu et al. were used [23]: CIDEA; Forward: CATGTATGAGATGTACTCCGTGTC, Reverse: GAGTAGGACAGGAACCGCAG (Gene Bank number NM_001279.3) (PCR product: 90 bp); for β-actin, used as internal control, they were 5′-AGGCCAACCGCGAGAAGATGACC-3′ (forward) and 5′-GAAGTCCAGGGCGACGTAGCAC-3′ (reverse); β-actin PCR product: 332 bp) (Gene Bank-Actin beta [NM_001101.3]) [24]. The gene-specific primers were purchased from Oligo™ Macrogen. The primer concentration is optimized for use in real-time PCR to determine the minimum primer concentration giving the lowest CT (threshold cycle) and minimizing nonspecific amplification.

##### Quantitative real-time PCR

The real-time PCR assays were performed with real-time PCR (Applied Biosystem 7500, USA) with 96-well plates, using a SybrGreen reagent [SYBR^®^ Green PCR Master Mix (Applied Biosystem, USA, Cat. No. 4344463)]. Reactions were performed in duplicates; each reaction (25 μl) contains forward and reverse primers (10 pmol), a 12.5 μl Power Sybr^®^ Green PCR master mix reaction buffer (Applied Biosystems), and 2 μl cDNA. The cycling parameters were 95 °C for 30 s, 60 °C for 1 min, and 40 cycles after an initial step of 95 °C for 10 min. The CT (cycle threshold) values were calculated. Melting curve analysis and agarose gel electrophoresis 2% were used to confirm the specificity of the PCR products. A reaction with no template was used as a negative control in each experiment.

The relative quantification of the CIDEA gene in different samples was calculated by a comparative method. β actin was used as an endogenous control. Each analysis was done with 4 reactions (2 for the target gene, CIDEA, and 2 for analysis of the internal control, β actin). The ΔCT for each sample was calculated, and finally the ΔΔCT between MS and control samples was calculated and linearized using 2^−ΔΔCt^ for overall change.

### DNA fragmentation analysis using comet assay

A modified alkaline Comet assay for sperm was carried out on the prepared samples according to the method of Hughes et al. [25]. Fully frosted slides were covered with 100 ml of 0.5% normal melting point agarose, and the agarose was allowed to solidify. Sperm in BWW (10 μl) were mixed with 90 μl of 0.5% low melting point agarose that was used to form the second layer. Then, the slides were placed in a lysis buffer for 1 h (2.5 M NaCl, 100 mMNaEDTA, 10 mMTris, 1% Triton X-100, and 10% dimethyl sulphoxide, pH 10). The slides were incubated overnight at 37 °C in 100 ml/ml proteinase K that had been added to the lysis buffer. Then, the slides were placed in a horizontal electrophoresis unit filled with fresh alkaline electrophoresis solution containing 300 mMNaOH and 1 mM EDTA, pH 12.5, for 20 min, to allow unwinding of the DNA in the cells. Electrophoresis was performed for 10 min at room temperature, at 2 V (0.714 V/cm) and 300 mA, obtained by adjusting the buffer level. The slides were then washed with a neutralizing solution of 0.4 M Tris, pH 7, to remove alkali and detergents. After neutralization, the slides were stained with ethidium bromide (20 mg/ml) and covered with a cover-slip. All steps were carried out under yellow light to prevent further DNA damage.

Each slide was examined under a 20× objective of a fluorescence microscope equipped with an excitation filter of 565 nm and a barrier filter of 590 nm. A digital camera was attached to the microscope, and images of the cells were processed by a computer-assisted image-analysis system (Comet Score, TriTek, USA) to determine the comet parameters. Fifty cells were scored from each replicate slide (100 cells in total). Results were expressed as tail length (TL; the distance that the DNA migrated) and also percentage of DNA in tail (% Tail; the density of the migrated DNA), one of the most reliable measurements for detecting DNA damage and tail moment (TM) (tail length × tail percentage of DNA/100).

### Homeostasis model assessment of insulin resistance (HOMA-IR)

In each sample, the degree of insulin resistance was estimated by the HOMA-IR as described by Matthews et al. [26]. HOMA-IR was calculated by taking into account the fasting insulin and blood glucose levels according to the equation (HOMA-IR) = fasting insulin (μU/ml) × fasting plasma glucose (mg/dl) × 0.0551/22.5. Serum and seminal insulin levels were estimated using the ELISA Kit [27]. The kit was provided by Diagnostic Systems Laboratories. Inc. Corporate Headquarters, 445 Medical Center Blvd., Webster, Texas 77598-4217. Seminal and fasting blood glucose levels were measured colorimetrically [7].

### Statistical analysis

Data are expressed as mean value ± SD. Comparisons are carried out by analysis of variance followed by Tukey’s test, using SPSS for Windows (17.0 Version). For statistical comparison between different groups, the significance of difference is tested using an ANOVA (analysis of variance) to compare results between more than two groups of numerical data. A Pearson correlation is used to assess relations between variables. Differences are considered statistically significant when P < 0.05.

## Results

**Fig. 1:**
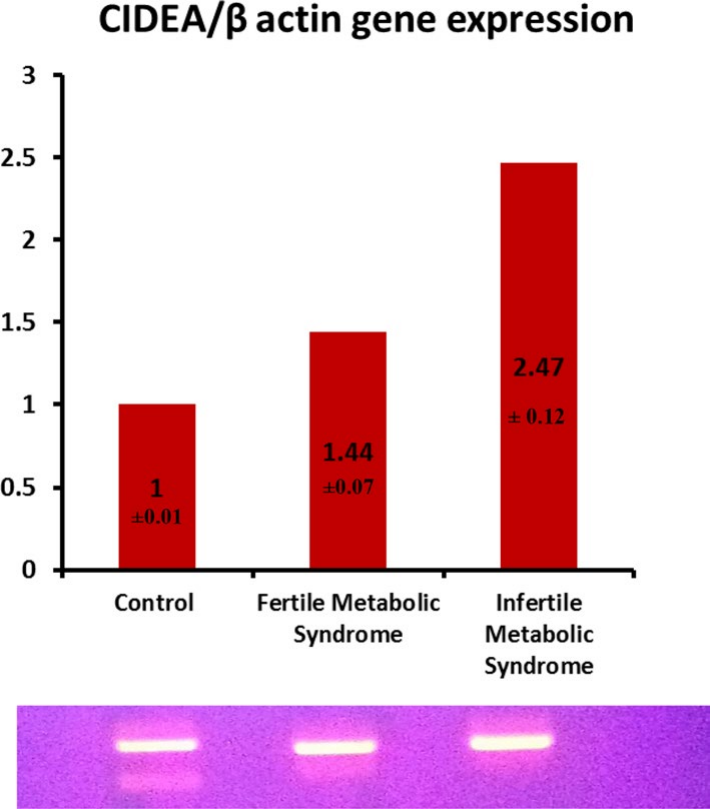
Agarose gel showing the RT-PCR product of gene expression of CIDEA gene in all of the studied groups and histogram representing the different gene expression values

**Fig. 2:**
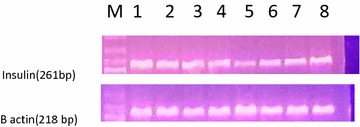
Agarose gel showing the RT-PCR product of gene expression of spermatozoal insulin gene in all of the studied groups. A representative photograph is shown for two control samples (*lanes 5*, *6*), three fertile metabolic syndrome samples (*lanes 4*, *7* and *8*) and three infertile metabolic syndrome samples (*1*, *2* and *3*). *M* corresponds to DNA ladder (100 bp)

Ages, some metabolic parameters, different semen parameters, and genetic studies of subjects and controls are supplied in Table 1. Spermatozoa insulin and CIDEA genes expression were significantly increased in the infertile MS group compared to the fertile MS group, and both were significantly higher than in the control group (Table 1; Figs. 1, 2).

**Table 1:** General, seminal and molecular parameters in the studied subjects

	Control (n = 45)	Fertile metabolic syndrome (n = 38)	Infertile metabolic syndrome (n = 37)	P
	Mean ± SD	Mean ± SD	Mean ± SD	
General parameters				
Age (years)	39.29 ± 8.52	40.01 ± 8.22	42.59 ± 7.47	0.17
BMI	23.64 ± 2.79	34.7 ± 8.1^a^	35.82 ± 4.37^a^	<0.001
HOMA-IR	1.70 ± 0.31	4.18 ± 0.90^a^	4.31 ± 0.95^a^	<0.001
Seminal parameters				
Volume (ml)	2.18 ± 0.54	2.37 ± 0.67	2.29 ± 0.56	0.33
Sperm concentration (10^6^/ml)	39.45 ± 14.20	37.78 ± 9.91	34.73 ± 9.69	0.19
Progressive motility; A + B (%)	49.67 ± 14.66	43.68 ± 11.24	32.05 ± 8.69^ab^	<0.001
Vitality (%)	68.70 ± 22.04	54.73 ± 16.14^a^	36.00 ± 8.45^ab^	<0.001
Normal morphology (%)	23.53 ± 6.78	22.44 ± 5.02	16.08 ± 4.29^ab^	<0.001
Seminal glucose (mg/dl)	33.39 ± 7.49	36.5 ± 6.68	41.73 ± 11.35^ab^	<0.001
Seminal insulin (μIU/L)	193.72 ± 52.91	302.28 ± 70.16^a^	477.83 ± 90.60^ab^	<0.001
Molecular parameters				
Insulin/β actin gene expression	0.94 ± 0.24	1.23 ± 0.25^a^	1.88 ± 0.63^ab^	<0.001
CIDEA/β actin gene expression	1.0 ± 0.01	1.44 ± 0.07^a^	2.47 ± 0.12^ab^	<0.001
DNA fragmentation (%)	20.78 ± 7.15	26.95 ± 9.43^a^	35.09 ± 12.50^ab^	<0.001
Tail length (μm)	0.76 ± 0.17	1.86 ± 0.42^a^	3.68 ± 0.76^ab^	<0.001
DNA percentage in tail (%)	6.37 ± 1.39	8.61 ± 2.64^a^	18.76 ± 3.72^ab^	<0.001
Tail Moment (TM)	1.11 ± 0.29	3.38 ± 0.98^a^	5.25 ± 1.25^ab^	<0.001

*SD* standard deviation, *P* calculated probability

P significant if <0.05

^a^Significant relative to control group

^b^Significant relative to fertile-metabolic syndrome group

Spermatozoa nDNA fragmentation, detected quantitatively by the Comet assay technique, shows a higher percentage in the infertile MS group than in the fertile MS group, and both MS groups are significantly higher than the control group. In addition, qualitative assessment of the degree of DNA fragmentation shows a similar result; these parameters are represented in Table 1 by tail length, DNA percentage in the tail, and tail moment.

**Fig. 3:**
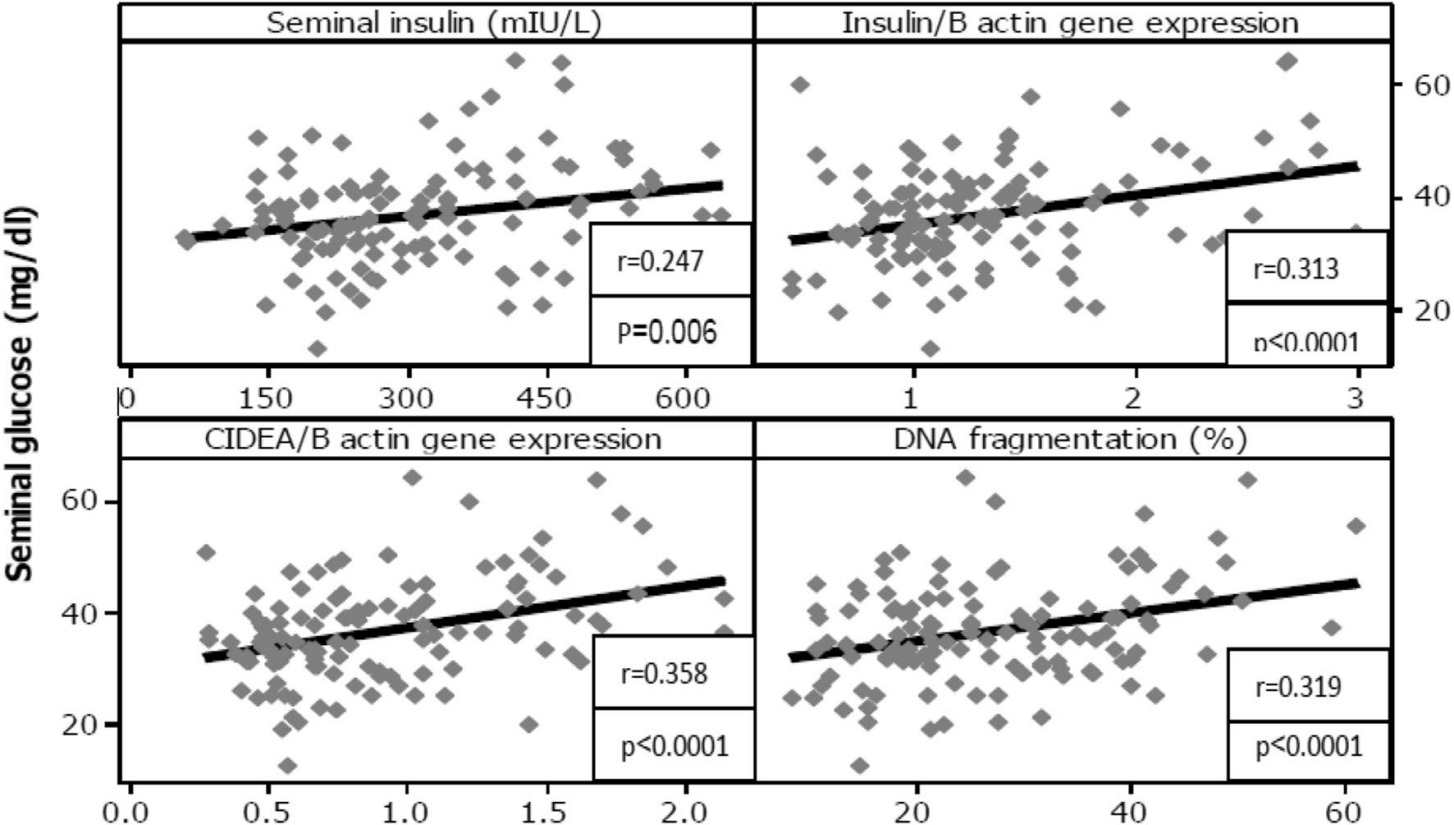
Correlations between seminal glucose concentration and seminal insulin level, spermatozoa insulin gene and CIDEA gene expressions as well as spermatozoa DNA fragmentation in all studied groups (n = 120). *r* Pearson correlation coefficient, *P* calculated probability

**Fig. 4:**
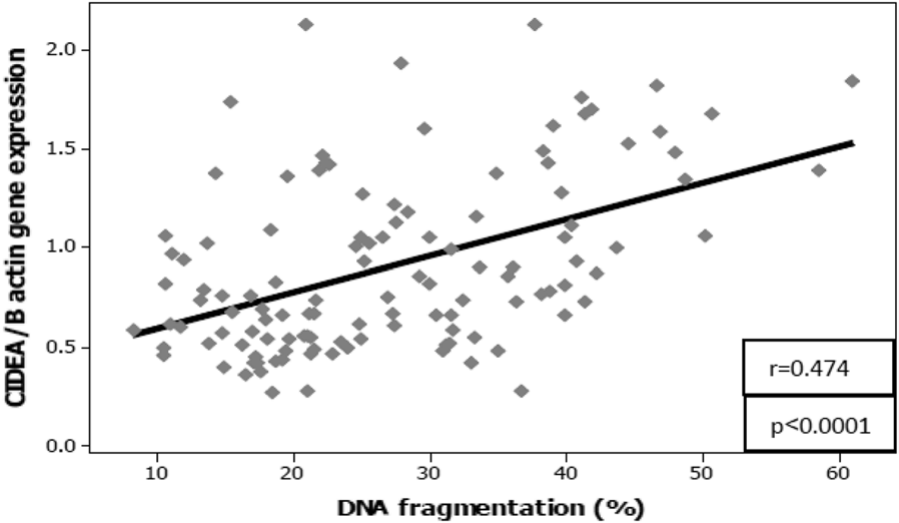
Correlation between spermatozoa CIDEA gene expression and spermatozoa DNA fragmentation in all studied groups (n = 120). *r* Pearson correlation coefficient, *P* calculated probability

In Fig. 3, the seminal glucose concentration shows a significant positive correlation with the seminal insulin level (r = 0.247; P = 0.006), spermatozoa insulin gene expression (r = 0.313; P < 0.0001), spermatozoa CIDEA gene expression (r = 0.358; P < 0.0001), and percentage of spermatozoa nDNA fragmentation (r = 0.319; P < 0.0001) in all studied groups (n = 120). Moreover, Fig. 4 shows a significant positive correlation between spermatozoa CIDEA gene expression and the percentage of sperm nDNA fragmentation (r = 0.474; P < 0.0001) in all studied groups (n = 120).

## Discussion

To our knowledge, the present work can be considered the first study to investigate the possible molecular mechanisms by which MS can affect male fertility. In this study, the patients included were suffering from male infertility despite a normal semen analysis, endocrine profile, and physical examination. The only suggested risk factor of infertility was having MS. A possible link between infertility and MS was also suggested by Juárez-Bengoa et al. [28], as they found a high prevalence (61.6%) of MS in a studied infertile population.

In our study, subjects with MS had significantly higher BMI and HOMA-IR in comparison to the controls, and this was expected as they had criteria of MS [16]. In addition, subjects and controls had normal semen parameters according to WHO recommendations [1]. However, there were significant decreases in progressive motility, sperm vitality, and normal morphology in the infertile MS group, in comparison with the fertile MS and control groups. Also, there was a significant decrease in vitality only in the fertile MS group in comparison with the control group. These results are in agreement with those of Leisegang et al. [6, 29] and Bhattacharya et al. [30]. Moreover, Agbaje et al. [11] reported nearly similar results but with no significant differences in any of the conventional sperm parameters and they concluded that the significant differences could be at the ‘molecular’ and not ‘cellular’ level. In addition, MacDonald et al. [31] found no relationship between obese/overweight status and semen parameters. As obesity is a fundamental constituent of MS, it was theorized that some of the significant findings in the obesity studies might be related to the underlying metabolic pathophysiology associated with MS [29].

On the other hand, several studies documented that obese/overweight status might result in hypogonadism; increased scrotal temperatures; direct negative effects on spermatogenesis, sperm functions, and sperm count; and increased sperm nDNA damage [5, 6, 32–34]. Regarding insulin resistance, significant decreases in the semen volume, total sperm count, percent motility, percent of rapid progressive motility, and percent normal morphology were found in diabetic males [30]. However, the values of these parameters were still within the normal ranges according to WHO criteria [1]. In addition, it was reported that dyslipidemia, as a component of MS, was associated with sperm abnormalities and might lead to the development of male infertility [4, 35, 36]. Finally, it was found that there was an inverse relationship between blood pressure and total serum testosterone concentrations, which might result in impaired reproductive potential [37].

In the current study, seminal insulin and spermatozoal insulin gene expression were significantly higher in the MS groups in comparison with the control group. In addition, they were significantly higher in the infertile MS group in comparison with the fertile MS group. Furthermore, seminal glucose was significantly higher in the infertile MS group in comparison with the fertile MS group and the control group. Seminal glucose concentration also showed a significant positive correlation with seminal insulin level and spermatozoa insulin gene expression. These results are supported by the work of Leisegang et al. [6]. They found increased serum and seminal insulin levels, with a positive correlation between them, in obese individuals. Also, Alves et al. [38] reported that the semen of diabetic men had higher fructose and glucose levels.

Insulin freely crosses the blood-testis barrier (BTB) [39] and can be secreted by the seminal vesicles [40]. Sertoli cells (SCs) also express, synthesize, and secrete insulin within the testes [41]. All of these mechanisms can explain the presence of seminal insulin in MS subjects. In addition, insulin is demonstrated to be synthesized by ejaculated spermatozoa with autocrine regulatory functions [20, 42]. In MS, insulin resistance in SCs may hypothetically be associated with a decrease in spermatogenesis. As increased seminal insulin is associated with insulin resistance and abdominal obesity, increased insulin exposure during spermatogenesis may potentially develop insulin resistance in the SCs and within the spermatozoa themselves. Evidence to support this hypothesis may be found in the intracellular molecular cascades associated with insulin receptor stimulation in these cells [6]. Insulin exerts its effect on spermatozoa via the PI3K/Akt intracelleular signaling pathway, leading to protein kinase B (PKB) phosphorylation, which may mediate beneficial effects on ejaculated spermatozoa [42].

In human tissues, this intracellular pathway is negatively influenced in insulin resistance. Hypothetically, over the spermatogenic cycle, it is conceivable that spermatozoa may develop insulin resistance in a manner similar to other tissue cells via a breakdown of the PI3K/Akt intracellular signaling pathway. This hypothesis would provide an explanation as to the potential negative association between increased seminal insulin and reduced motility of ejaculated sperm [6]. Reactive oxygen species (ROS) production stimulated by increased glucose may have a role in the pathogenesis of insulin resistance [38].

The autocrine synthesis of insulin by ejaculated spermatozoa with subsequent increase in seminal insulin can be considered as a feedback or defense mechanism against accumulated unutilized seminal glucose, leading to the increase in seminal insulin and glucose levels. This can clarify the positive correlation between seminal glucose and seminal insulin levels, and the significant increase in spermatozoa insulin gene expression in the infertile MS group with a significant positive correlation with seminal glucose.

In the present study, spermatozoa nDNA fragmentation and CIDEA gene expression were significantly higher in infertile MS patients than in fertile MS patients, and both were significantly higher than the control group. In addition, qualitative assessment of the degree of DNA fragmentation shows similar results represented by tail length, DNA percentage in the tail, and tail moment. Moreover, seminal glucose concentration showed a significant positive correlation with spermatozoa CIDEA gene expression and the percentage of spermatozoa nDNA fragmentation. Also, a significant positive correlation between spermatozoa CIDEA gene expression and percentage of sperm nDNA fragmentation was detected. This is in agreement with Leisegang and colleagues [29], who found significantly higher percentages of spermatozoa with disturbed mitochondrial membrane potential and DNA damage in MS patients. Obesity, hyperglycemia, and dyslipidemia were reported to induce oxidative stress state in the testicular microenvironment leading to increase production of ROS [4, 6, 30, 43–46]. As human spermatozoa are highly sensitive to oxidative stress, sperm plasma membrane damage and nuclear or mitochondrial DNA fragmentation can occur in response to ROS [47]. Significant DNA fragmentation can present in normozoospermic subfertile men, so it may be an important mechanism of MS associated subfertility [48]. In addition to increased spermatozoa ROS production in MS, decreased ATP synthesis [49] and systemic inflammation [50–52] can contribute of increased sperm nDNA fragmentation in MS.

Increased spermatozoa CIDEA gene expression in our study appeared to be a sequence of sperm plasma membrane fatty acids oxidation. It was reported that patients with high fatty acids oxidation ability showed a higher CIDEA mRNA gene expression. CIDEA is important for the regulation of lipolysis, and disturbance in its expression may be responsible for metabolic complications of MS [15]. They also, reported that transient overexpression of CIDEA gene is associated with increased oxidation of fatty acids and diminished oxidation of glucose. They observed in their study that glucose oxidation is 40% decreased in response to the in the enhanced CIDEA expression [15]. It may be induced as a defense mechanism to protect against energy depletion in the spermatozoa of MS patients [53, 54]. As both DNA fragmentation and CIDEA gene expression could be due to the same causes and happen in the same circumstances, a positive correlation between them appears reasonable, and both of them could be reliable markers for sperm functions and spermatozoa oxidative stress states. In addition, there were positive correlations between seminal glucose and DNA fragmentation, as well as CIDEA gene expression, in all the studied groups’ subjects collectively. Increased sperm DNA damage was reported in diabetic men due to high oxidative stress as a direct consequence of increased glycolytic capacity, induced by higher glucose availability in semen [38]. Amplified oxidative stress and higher DNA fragmentation are interconnected with apoptosis [55], which has been reported to be increased in spermatozoa from type 1 diabetes mellitus and type 2 diabetes mellitus patients [56].

Hyperglycemia and increased seminal glucose levels accelerate the accumulation of advanced glycation end products (AGEs), e.g. carboxymethyllysine, which are capable of producing, promoting, and/or intensifying oxidative stress and induction of sperm nDNA damage [57]. The detection of high levels of advanced glycation end products (AGEs) and their receptors (RAGEs) throughout the male reproductive tract, coupled with changes in testicular metabolite levels and spermatogenic gene expression, suggested that glycation might play an integral role in oxidative stress, which in turn damages sperm DNA [11, 58].

There are some limitations to the present study. The most important limitation is the lack of objective data supporting the proposed pathophysiology pathway of spermatozoal DNA fragmentation in the studied subjects, as data on ROS levels for the studied subgroups was lacking. We recommend repeating this study to find a correlation between spermatozoal DNA fragmentation and seminal ROS levels. The small number of included subjects was another limitation. So, it is recommended to repeat this study with a larger number of subjects, to confirm or disconfirm its findings. In addition, further investigations are needed for comparison, interpretation, and defining the demarcating step at which MS can actually be considered as an etiologic factor for male infertility.

## Conclusions

It could be concluded that MS may play a pivotal role in affecting male fertility potential at the molecular level, through its possible inducing effect on the pro-apoptotic genes like the CIDEA gene. The role of CIDEA is mediated through its effect on spermatozoa nDNA fragmentation as well as insulin gene expression, which cannot be detected by the conventional computer-assisted semen analysis techniques. Moreover, the results of this study point to a new spermatozoa stress condition: spermatozoa insulin resistance state as a part of insulin resistance syndrome (MS). Spermatozoa insulin resistance state is manifested by increased spermatozoa insulin gene expression, as well as increased seminal plasma insulin and glucose levels. Consequently, MS can be considered as a stress factor that affects spermatozoa through increased seminal plasma insulin and glucose levels.

## Abbreviations

CIDEA: spermatozoal cell death-inducing DNA fragmentation factor-α-like effector A
MS: metabolic syndrome
RT-PCR: real time-polymerase chain reaction
HOMA-IR: homeostasis model assessment of insulin resistance
WHO: World Health Organization
AGEs: advanced glycation end products
nDNA: nuclear DNA
CDU: Doppler ultrasounds
CASA: computer assisted semen analysis
DEPC: diethyl pyrcarbonate
PKB: protein kinase B
ROS: reactive oxygen species
RAGEs: receptors of advanced glycation end products

## References

[1] World Health Organization (2010). WHO laboratory manual for the examination and processing of human semen.

[2] Jarow JP, Sharlip ID, Belker AM, Lipshultz LI, Sigman M, Thomas AJ, Schlegel PN, Howards SS, Nehra A, Damewood MD, Overstreet JW, Sadovsky R (2010). Male infertility best practice policy committee of the American Urological Association Inc: best practice policies for male infertility. J Urol.

[3] Taslim S, Tai ES (2009). The relevance of the metabolic syndrome. Ann Acad Med Singap.

[4] Kasturi SS, Tannir J, Brannigan RE (2008). The metabolic syndrome and male infertility. J Androl.

[5] Du Plessis SS, Cabler S, McAlister DA, Sabanegh E, Agarwal A (2010). The effect of obesity on sperm disorders and male infertility. Nat Rev Urol.

[6] Leisegang K, Bouic PJ, Menkveld R, Henkel RR (2014). Obesity is associated with increased seminal insulin and leptin alongside reduced fertility parameters in a controlled male cohort. Reprod Biol Endocrinol.

[7] Truta Z, Garlovanu M, Lerintiu S, Micu R (2010). A new method for human semen glucose concentration evaluation. Rom Biotech Lett.

[8] Mallidis C, Czerwiec A, Filippi S, O’Neill J, Maggi M, McClure N (2011). Spermatogenic and sperm quality differences in an experimental model of metabolic syndrome and hypogonadal hypogonadism. Reproduction.

[9] Chi HJ, Chung DY, Choi SY, Kim JH, Kim GY, Lee JS, Lee HS, Kim MH, Roh SI (2011). Integrity of human sperm DNA assessed by the neutral comet assay and its relationship to semen parameters and clinical outcomes for the IVF-ET program. Clin Exp Reprod Med.

[10] Oleszczuk K, Augustinsson L, Bayat N, Giwercman A, Bungum M (2013). Prevalence of high DNA fragmentation index in male partners of unexplained infertile couples. Andrology.

[11] Agbaje IM, Rogers DA, McVicar CM, McClure N, Atkinson AB, Mallidis C, Lewis SE (2007). Insulin dependent diabetes mellitus: implications for male reproductive function. Hum Reprod.

[12] Nordstrom EA, Ryden M, Backlund EC, Dahlman I, Kaaman M, Blomqvist L, Cannon B, Nedergaard J, Arner P (2005). A human-specific role of cell death-inducing DFFA (DNA fragmentation factor-α)-like effector A (CIDEA) in adipocyte lipolysis and obesity. Diabetes.

[13] Zhang L, Dai Y, Bian L, Wang W, Wang W, Muramatsu M, Hua Q (2011). Association of the cell death-inducing DNA fragmentation factor alpha-like effector A (CIDEA) gene V115F (G/T) polymorphism with phenotypes of metabolic syndrome in a Chinese population. Diabetes Res Clin Pract.

[14] Gummesson A, Jernas M, Svensson PA (2007). Relations of adipose tissue CIDEA gene expression to basal metabolic rate, energy restriction, and obesity: population-based and dietary intervention studies. J Clin Endocrinol Metab.

[15] Laurencikiene J, Stenson BM, Nordstrom EA, Agustsson T, Langin D, Isaksson B, Permert J, Ryden M, Arner P (2008). Evidence for an important role of CIDEA in human cancer cachexia. Cancer Res.

[16] Alberti KG, Eckel RH, Grundy SM, Zimmet PZ, Cleeman JI, Donato KA, Fruchart JC, James WP, Loria CM, Smith SC (2009). Harmonizing the metabolic syndrome: a joint interim statement of the International Diabetes Federation Task Force on Epidemiology and Prevention; National Heart, Lung, and Blood Institute; American Heart Association; World Heart Federation; International Atherosclerosis Society; and International Association for the Study of Obesity. Circulation.

[17] Politch JA, Tucker L, Bowman FP, Anderson DJ (2007). Concentrations and significance of cytokines and other immunologic factors in semen of healthy fertile men. Hum Reprod.

[18] Endtz AW (1974). A rapid staining method for differentiating granulocytes from “germinal cells” in Papanicolaou-stained semen. Acta Cytol.

[19] Björndahl L, Söderlund I, Kvist U (2003). Evaluation of the one-step eosin-nigrosin staining technique for human sperm vitality assessment. Hum Reprod.

[20] Aquila S, Gentile M, Middea E, Catalano S, Andò S (2005). Autocrine regulation of insulin secretion in human ejaculated spermatozoa. Endocrinology.

[21] Raha S, Ling M, Merante F, Replay R, Walker JM (1998). Extraction of total RNA from tissues and cultured cells. Molecular biomethods handbook.

[22] Zumárraga M, Andía I, Dávila R, Miller JC, Friedhoff AJ (2004). Expression in normals and in subjects with schizophrenia of a novel gene fragment originally isolated from monozygotic twins discordant for schizophrenia. Genet Mol Biol.

[23] Yu M, Wang H, Zhao J, Yuan Y, Wang C, Li J, Zhang L, Zhang L, Li Q, Ye J (2013). Expression of CIDE proteins in clear cell renal cell carcinoma and their prognostic significance. Mol Cell Biochem.

[24] Dupasquier S, Delmarcelle AS, Marbaix E, Cosyns JP, Courtoy PJ, Pierreux CE (2014). Validation of housekeeping gene and impact on normalized gene expression in clear cell renal cell carcinoma: critical reassessment of YBX3/ZONAB/CSDA expression. BMC Mol Biol.

[25] Hughes CM, McKelvey-Martin VJ, Lewis SE (1999). Human sperm DNA integrity assessed by the Comet and ELISA assays. Mutagenesis.

[26] Matthews DR, Hosker JP, Rudenski AS, Naylor BA, Treacher DF, Turner RC (1985). Homeostasis model assessment: insulin resistance and b-cell function from fasting plasma glucose and insulin concentration in man. Diabetologia.

[27] Hwang DL, Barsenghian G, Lev-Ran A (1985). Determination of free insulin in antibody containing sera: comparison of polyethylene glycol and *staphylococcous* aureus cells. Horm Metab Res.

[28] Juárez-Bengoa A, Rodríguez-Perdomo D, Pizano-Zárate ML (2011). Hypogonadism associated with metabolic syndrome in infertile men. Rev Mex Urol.

[29] Leisegang K, Udodong A, Bouic PJD, Henkel RR (2014). Effect of the metabolic syndrome on male reproductive function: a case-controlled pilot study. Andrologia.

[30] Bhattacharya SM, Ghosh M, Nandi N (2014). Diabetes mellitus and abnormalities in semen analysis. J Obstet Gynaecol Res.

[31] MacDonald AA, Herbison GP, Showell M, Farquhar CM (2010). The impact of body mass index on semen parameters and reproductive hormones in human males: a systematic review with meta-analysis. Hum Reprod Update.

[32] Hammoud AO, Gibson M, Peterson CM, Meikle AW, Carrell DT (2008). Impact of male obesity on infertility: a critical review of the current literature. Fertil Steril.

[33] Sermondade N, Faure C, Fezeu L, Levy R, Czernichow S (2012). Obesity and increased risk for oligozoospermia and azoospermia. Arch Intern Med.

[34] Sermondade N, Faure C, Fezeu L, Shayeb AG, Bonde JP (2013). BMI in relation to sperm count: an updated systematic review and collaborative meta-analysis. Hum Reprod Update.

[35] Ramirez-Torres MA, Carrera A, Zambrana M (2000). High incidence of hyperestrogenemia and dyslipidemia in a group of infertile men. Ginecol Obstet Mex.

[36] Shalaby MA, el-Zorba HY, Kamel GM (2004). Effect of alphatocopherol and simvastatin on male fertility in hypercholesterolemic rats. Pharmacol Res.

[37] Torkler S, Wallaschofski H, Baumeister SE, Völzke H, Dörr M, Felix S, Rettig R, Nauck M, Haring R (2011). Inverse association between total testosterone concentrations, incident hypertension and blood pressure. Aging Male.

[38] Alves MG, Martins AD, Rato L, Moreira PI, Oliveira PF, Socorro S (2013). Molecular mechanisms beyond glucose transport in diabetes-related male infertility. Biochim Biophys Acta.

[39] García-Díez LC, Corrales Hernandez JJ, Hernandez-Diaz J, Pedraz MJ, Miralles JM (1991). Semen characteristics and diabetes mellitus: significance of insulin in male infertility. Arch Androl.

[40] Stahler MS, Budd GC, Pansky B (1987). Evidence for insulin synthesis in normal mouse seminal vesicle based on in situ RNA-DNA hybridization. Biol Reprod.

[41] Schoeller EL, Albanna G, Frolova AI, Moley KH (2012). Insulin rescues impaired spermatogenesis via the hypothalamic-pituitary-gonadal axis in Akita diabetic mice and restores male fertility. Diabetes.

[42] Andò S, Aquila S (2005). Arguments raised by the recent discovery that insulin and leptin are expressed in and secreted by human ejaculated spermatozoa. Mol Cell Endocrinol.

[43] Chavarro JE, Toth TL, Wright DL, Meeker JD, Hauser R (2010). Body mass index in relation to semen quality, sperm DNA integrity, and serum reproductive hormone levels among men attending an infertility clinic. Fertil Steril.

[44] Fariello RM, Pariz JR, Spaine DM, Cedenho AP, Bertolla RP, Fraietta R (2012). Association between obesity and alteration of sperm DNA integrity and mitochondrial activity. BJU Int.

[45] Dupont C, Faure C, Sermondade N, Boubaya M, Eustache F, Clement P, Briot P, Berthaut I, Levy V, Cedrin-Durnerin I, Benzacken B, Chavatte-Palmer P, Levy R (2013). Obesity leads to higher risk of sperm DNA damage in infertile patients. Asian J Androl.

[46] Palmer NO, Bakos HW, Fullston T, Lane M (2012). Impact of obesity on male fertility, sperm function and molecular composition. Spermatogenesis.

[47] Aitken RJ, Baker MA (2006). Oxidative stress, sperm survival and fertility control. Mol Cell Endocrinol.

[48] Wright C, Milne S, Leeson S (2014). Sperm DNA damage caused by oxidative stress: modifiable clinical, lifestyle and nutritional factors in male infertility. Reprod Biomed Online.

[49] Marchetti P, Ballot C, Jouy N, Thomas P, Marchetti C (2012). Influence of mitochondrial membrane potential of spermatozoa on in vitro fertilization outcome. Andrologia.

[50] Furukawa S, Fujita T, Shimabukuro M, Iwaki M, Yamada Y, Nakajima Y, Nakayama O, Makishima M, Matsuda M, Shimomura I (2004). Increased oxidative stress in obesity and its impact on metabolic syndrome. J Clin Invest.

[51] Henkel R (2005). The impact of oxidants on sperm function. Andrologia.

[52] Martinez R, Proverbio F, Camejo MI (2007). Sperm lipid peroxidation and pro-inflammatory cytokines. Asian J Androl.

[53] Johansson LE, Danielsson APH, Parikh H, Klintenberg M, Norstrom F, Groop L, Ridderstrale M (2012). Differential gene expression in adipose tissue from obese human subjects during weight loss and weight maintenance. Am J Clin Nutr.

[54] Yu T, Robotham JL, Yoon Y (2006). Increased production of reactive oxygen species in hyperglycemic conditions requires dynamic change of mitochondrial morphology. Proc Natl Acad Sci.

[55] Aitken RJ, Koppers AJ (2011). Apoptosis and DNA damage in human spermatozoa. Asian J Androl.

[56] Roessner C, Paasch U, Kratzsch J, Glander HJ, Grunewald S (2012). Sperm apoptosis signalling in diabetic men. Reprod Biomed Online.

[57] Mallidis C, Agbaje IM, Rogers DA, Glenn JV, Pringle R, Atkinson AB, Steger K, Stitt AW, McClure N (2009). Advanced glycation end products accumulate in the reproductive tract of men with diabetes. Int J Androl.

[58] La Vignera S, Condorelli R, Vicari E, D’Agata R, Calogero AE (2012). Diabetes mellitus and sperm parameters. J Androl.

